# Editorial: Meta-omic Approaches to the Complex Anaerobic Communities in Wastewater Treatment Plants and Digesters

**DOI:** 10.3389/fmicb.2021.664716

**Published:** 2021-05-07

**Authors:** Leonardo Erijman, Stefano Campanaro, Claudia Etchebehere, Maria Bernadete Amancio Varesche

**Affiliations:** ^1^Instituto de Investigaciones en Ingeniería Genética y Biología Molecular “Dr Héctor N. Torres” (INGEBI-CONICET), Buenos Aires, Argentina; ^2^Departamento de Fisiología, Biología Molecular y Celular, Facultad de Ciencias Exactas y Naturales, Universidad de Buenos Aires, Buenos Aires, Argentina; ^3^Department of Biology, University of Padua, Padua, Italy; ^4^Centro di Ricerca Interdipartimentale per le Biotecnologie Innovative (CRIBI), University of Padua, Padua, Italy; ^5^Instituto de Investigaciones Biológicas Clemente Estable (IIBCE), Montevideo, Uruguay; ^6^Laboratório de Processos Biológicos, Departamento de Hidráulica e Saneamento, Escola de Engenharia de São Carlos, Universidade de São Paulo, São Paulo, Brazil

**Keywords:** anaerobic digestion, biogas microbiome, metagenomics, metaproteomics, environmental biotechnology

Anaerobic digestion is an established process for the stabilization of organic matter and production of renewable energy that has a central role in sustainable development and circular economy. It is currently being used worldwide for fuel generation and the production of a stabilized fertilizer from livestock manures and crop residues. High-rate anaerobic technologies can be applied to treat wastewater from virtually any industrial process, and novel strategies open avenues for obtaining products of biotechnological interest, including alternative energy carriers, such as methane and hydrogen, and valuable end-products with industrial application, such as organic acids.

The anaerobic digestion process is driven by a complex network of specialized microbial guilds. Environmental and operational factors, as well as immigration and stochastic events, determine the microbial community structure of anaerobic digesters. These communities consist of hundreds of different species of bacteria and archaea, together making up the “biogas microbiome” (Campanaro et al., 2020; Nierychlo et al., 2020). Only a minor fraction of the microbiome is accessible by cultivation-based approaches, which are additionally unable to capture complex interactions. Therefore, meta-omic techniques are key to gain deeper understanding of the functional importance of specific organisms and to elucidate microbial interactions such as metabolic cross-feeding.

The five articles in this Research Topic asked relevant questions about the roles of individual or defined populations of microbes in the anaerobic digestion process and used metagenomic and metatranscriptomic tools to address those questions.

Mei et al. revealed the role of previously uncharacterized Bacteroidales populations as key players for amino-acid fermentation. The authors combined short-term enrichment cultures with genome-centric metagenomic and metatranscriptomic resolution to unravel the pathway of electron transfer and energy conservation in this group of microbes. By mapping the sequences of the metagenome-assembled genomes to public metagenomic and metatranscriptomic datasets, Mei et al. found that members of the proposed new family of Bacteroidales appear to be abundant and widespread amino acid degraders in full-scale anaerobic digesters.

Chloroflexi are prominent members of the biogas microbiome. Based on the high abundance of members of Chloroflexi in anaerobic digesters, it is assumed that members of this phylum contribute significantly to the overall function of AD systems, presumably as primary fermenters (Petriglieri et al., 2018). Bovio-Winkler et al. compared reactors fed with either wastewater or solid waste in a meta-analysis of 62 full-scale methanogenic reactors and concluded that members of the class Anaerolineae predominate in anaerobic bioreactors. These microorganisms may have a structural role as their filamentous morphology would assist in maintaining granule structure. This work is a good example showing the potential of using sequence data deposited in databases to answer important questions about AD microbiomes.

The centrality of lactate cross-feeding in well-performing hydrogen producing reactors was illustrated by Detman et al. in the digestion of sugar-beet molasses in packed bed reactors. Optimal hydrogen production required a balance between hydrogen-producing bacteria (HPB), namely *Clostridium, Ruminococcus* and *Prevotella* and lactic acid bacteria (LAB), such as *Lactobacillus, Bifidobacterium, Leuconostoc, Streptococcus*, and *Lactococcus*. Surveys of the ratio between HPB and LAB reported in other hydrogen-producing microbial communities validated this conclusion. Intriguingly, Detman et al. found a strong correlation between *Caproiciproducens* and hydrogen production. *Caproiciproducens*, which was present at relatively high abundance, includes species involved in caproic acid biosynthesis from several electron donors, including lactate (Contreras-Dávila et al., 2020).

The growth and activity of specific microorganisms as well as the interaction between different microbial guilds may be severely affected by the occurrence of antimicrobials in anaerobic digesters. In their review article, Xiao et al. analyzed the impact of antibiotics on fermentative bacteria and methanogenic archaea reporting both negative and positive effects at different levels of the anaerobic digestion food chain. Authors reported the role of a range of antibiotics including macrolides, tetracyclines, β-lactams, and they also considered the impact of antibiotic mixtures. Analysis of scientific literature allowed the authors to summarize the influence of antibiotics on the anaerobic digestion process and to delineate some general “rules” about the short/long term effects, the duration of the bacteriostatic activity and the role of environmental factors in increasing or decreasing their impact.

Kim et al. evaluated the effects of inoculum heat shock, pH, temperature and solids retention time (SRT) on anaerobic digestion of waste activated sludge (WAS). They inferred the role of individual microbial populations from the net growth rates estimated using a mass balance, which considered 16S rRNA gene abundance in the influent and effluent. A major strength of the approach is that it could help discriminate populations that grow actively and those that are introduced by immigration with the feed sludge.

We still lack a complete understanding of community assembly that would allow us to move into the design of fully active synthetic communities. It is conceivable that species diversity and functional redundancy are critical to maintain stable process functioning. Meta-omic approaches are starting to uncover the numerous direct and indirect interactions that operate through networks of metabolic pathways. The new knowledge afforded by the articles in this topic represents another step forward in the understanding of the role of key microbial players on performance, stability, and versatility of the anaerobic digestion process.

## Author Contributions

All authors contributed to the preparation of this editorial.

## Conflict of Interest

The authors declare that the research was conducted in the absence of any commercial or financial relationships that could be construed as a potential conflict of interest.
